# No differences in subjective knee function between surgical techniques of anterior cruciate ligament reconstruction at 2-year follow-up: a cohort study from the Swedish National Knee Ligament Register

**DOI:** 10.1007/s00167-017-4521-y

**Published:** 2017-03-17

**Authors:** Eric Hamrin Senorski, David Sundemo, Christopher D. Murawski, Eduard Alentorn-Geli, Volker Musahl, Freddie Fu, Neel Desai, Anders Stålman, Kristian Samuelsson

**Affiliations:** 1 Department of Health and Rehabilitation, Institute of Neuroscience and Physiology, The Sahlgrenska Academy, University of Gothenburg, Göteborg, Sweden; 2 Department of Orthopaedics, Institute of Clinical Sciences, The Sahlgrenska Academy, University of Gothenburg, Göteborg, Sweden; 3 Department of Orthoapedic Surgery, University of Pittsburgh School of Medicine, Pittsburgh, PA USA; 4 Fundación García-Cugat, Barcelona, Spain; 5 Artroscopia GC, SL, Barcelona, Spain; 6 Mutualidad Catalana de Futbolistas – Delegación Cataluña, Federación Española de Fútbol, Barcelona, Spain; 7 Department of Orthopaedic Surgery, Mayo Clinic, Rochester, MN USA; 8 Department of Orthopaedics, Sahlgrenska University Hospital, Mölndal, Sweden; 9 Department of Molecular Medicine and Surgery, Stockholm Sports Trauma Research Center, Karolinska Institutet, Stockholm, Sweden

**Keywords:** Register, Anterior cruciate ligament, ACL, KOOS, Anatomic, Checklist, Patient-reported outcome

## Abstract

**Purpose:**

The purpose of this study was to investigate how different techniques of single-bundle anterior cruciate ligament (ACL) reconstruction affect subjective knee function via the Knee injury and Osteoarthritis Outcome Score (KOOS) evaluation 2 years after surgery. It was hypothesized that the surgical techniques of single-bundle ACL reconstruction would result in equivalent results with respect to subjective knee function 2 years after surgery.

**Methods:**

This cohort study was based on data from the Swedish National Knee Ligament Register during the 10-year period of 1 January 2005 through 31 December 2014. Patients who underwent primary single-bundle ACL reconstruction with hamstrings tendon autograft were included. Details on surgical technique were collected using a web-based questionnaire comprised of essential AARSC items, including utilization of accessory medial portal drilling, anatomic tunnel placement, and visualization of insertion sites and landmarks. A repeated measures ANOVA and an additional linear mixed model analysis were used to investigate the effect of surgical technique on the KOOS_4_ from the pre-operative period to 2-year follow-up.

**Results:**

A total of 13,636 patients who had undergone single-bundle ACL reconstruction comprised the study group for this analysis. A repeated measures ANOVA determined that mean subjective knee function differed between the pre-operative time period and at 2-year follow-up (*p* < 0.001). No differences were found with respect to the interaction between KOOS_4_ and surgical technique or gender. Additionally, the linear mixed model adjusted for age at reconstruction, gender, and concomitant injuries showed no difference between surgical techniques in KOOS_4_ improvement from baseline to 2-year follow-up. However, KOOS_4_ improved significantly in patients for all surgical techniques of single-bundle ACL reconstruction (*p* < 0.001); the largest improvement was seen between the pre-operative time period and at 1-year follow-up.

**Conclusion:**

Surgical techniques of primary single-bundle ACL reconstruction did not demonstrate differences in the improvement in baseline subjective knee function as measured with the KOOS_4_ during the first 2 years after surgery. However, subjective knee function improved from pre-operative baseline to 2-year follow-up independently of surgical technique.

**Electronic supplementary material:**

The online version of this article (doi:10.1007/s00167-017-4521-y) contains supplementary material, which is available to authorized users.

## Introduction

Optimizing long-term outcomes after anterior cruciate ligament (ACL) injury remains a challenge for both physicians and physical therapists. Although good results are reported across the literature, numerous studies highlight sub-optimal results and areas of improvement, including knee function [22, 25], return to sport [4, 14], as well as quality of life and development of osteoarthritis [9, 11]. Patient-reported outcome measures (PROMs) should be utilized to highlight the patient’s perspective on treatment outcome and represent the cornerstone in evaluating the success of intervention [24, 32]. In the case of ACL reconstruction, the Knee injury and Osteoarthritis Outcome Score (KOOS) evaluates subjective knee function and is one of the most frequently reported in the literature [13, 31].

The goals of ACL reconstruction are to restore the anatomy as closely as possible to the native knee, reestablish both biological and biomechanical functions, and prevent the development and/or progression of osteoarthritis. Over the last several decades, the surgical techniques for single-bundle ACL reconstruction have evolved and the traditional transtibial drilling technique has recently come under scrutiny. In this regard, it has been shown that transtibial drilling has the tendency to result in a non-anatomic reconstruction when evaluated in reference to the native ACL footprints [20, 34]. In comparison, anatomic reconstruction techniques, predominantly involving transportal tunnel drilling, have demonstrated superior results in both biomechanical and clinical studies when compared to non-anatomic techniques [18, 36]. However, several studies have suggested that grafts placed anatomically are exposed to greater (i.e. native) in situ forces as opposed to those placed non-anatomically [3, 23, 35]. In this regard, a study from the Danish Knee Ligament Reconstruction Register reported an increased risk of revision ACL surgery when a transportal technique was compared to reconstructions performed using a transtibial technique [28]. There are conflicting results in treatment outcome with regard to surgical factors in previous studies. However, most studies only investigate single surgical factors in a limited cohort.

Recently, the anatomic anterior cruciate ligament reconstruction scoring checklist (AARSC) was published as a tool to evaluate anatomic ACL reconstruction. This tool provides the opportunity to study how detailed knowledge regarding the surgical procedure of ACL reconstruction can affect treatment outcome. For instance, anatomic ACL surgery, characterized by the presence of essential AARSC items, was associated with a lower risk of revision surgery compared with anatomic bone tunnel placement via transportal drilling [7]. However, the potential association between AARSC and PROMs has not yet been studied and it therefore remains unknown as to how recovery in subjective knee function is affected by the surgical techniques of single-bundle ACL reconstruction.

The aim of this study was to investigate how different surgical techniques of single-bundle ACL reconstruction affect subjective knee function during the first 2 years after ACL reconstruction. The secondary aim was to compare subjective knee function stratified by surgical technique of single-bundle reconstruction pre-operatively, and at 1 and 2 years of follow-up. It was hypothesized that the surgical techniques of single-bundle ACL reconstruction would result in equivalent results with respect to subjective knee function 2 years after surgery.

## Materials and methods

### Participants

On 25 November 2015, patient data were extracted from the Swedish National Knee Ligament Register (SNKLR). Inclusion was set to patients aged 13–49 years who underwent primary single-bundle ACL reconstruction with hamstrings tendon (HT) autograft in the 10-year period ranging from 1 January 2005 to 31 December 2014. The follow-up time period was initiated at the date of primary ACL reconstruction and ended at the 2-year follow-up. Patients who underwent revision ACL surgery before the 2-year follow-up period were excluded. Patients were also excluded if information on the exact date for the index or revision ACL reconstruction, or details of the surgeon who performed the procedure were missing. The inclusion and exclusion criteria are summarized in Table 1.

**Table 1:** Summary of inclusion and exclusion criteria

*Inclusion criteria*
Primary ACL reconstruction
ACL reconstruction using hamstring tendon autograft
Single-bundle ACL reconstruction
Age 13–49 years Year of surgery 2005–2013
*Exclusion criteria*
Concomitant ligament injury requiring repair/reconstruction
Concomitant fracture/tendon injury
Concomitant vascular injury
Early contralateral ACL or revision surgery, within 550 days of index surgery

*ACL* anterior cruciate ligament, *KOOS* Knee injury and Osteoarthritis Outcome Score

### The Swedish National Knee Ligament Register

The SNKLR is a nationwide database that collects prospective data on ACL injuries and associated knee surgery. The registry utilizes a web-based protocol consisting of two parts: one surgeon-reported section and one patient-reported section. The surgeon-reported section includes information regarding the patients’ activity at the time of injury, time from injury to reconstruction, graft selection, fixation techniques, and previous surgery. The surgeon registers all surgical procedures on the injured knee, including concomitant injuries and treatment of the menisci and/or cartilage. The patient-reported section includes two PROMs:

- *Knee injury and Osteoarthritis Outcome Score* [31], for subjective functional knee-related outcome. The KOOS has high test–retest reliability for patients with knee injuries. The ICC has been described as 0.85–0.93 for the sub-scale of pain, 0.83–0.95 for the sub-scale of symptoms, 0.75–0.91 for the sub-scale of function in daily activities, 0.61–0.89 for the sub-scale of function in sport and recreation, and 0.83–0.95 for the sub-scale knee-related QoL [2]. The minimal important change in KOOS is considered to be 8–10 points for all sub-scales [19].
- *European Quality of Life*-*Five Dimension* [26], for health-related quality of life.

The SNKLR has reported a coverage (proportion of participating units in relation to all eligible units) of 92.9% and completeness (proportion of target population in the registry) >90%, with a 50–70% response rate for the patient-reported outcome measures [10]. Additionally, a non-response analysis has been performed, showing that the register is valid despite the sub-optimal number of patients responding at follow-up [29].

### Surgical techniques of single-bundle ACL reconstruction

To evaluate surgical technique, a web-based questionnaire was created to collect detailed information from ACL surgeons in Sweden. The questionnaire included items from the AARSC. The AARSC has been tested for validity and reliability, and consists of 17 items covering surgical technique and 1 item relating to documentation of bone tunnel placement. The checklist allows for calculation of an ‘anatomic score’ with a total of 19 points [6].

Each item in the questionnaire contains a two-part specified response: first, surgeons were asked whether they ‘Always’ or ‘Never’ used the surgical technique; second, surgeons were asked whether they still performed the surgical technique today. A time interval of identified surgical techniques was created for each surgeon who responded. For the study, it was necessary to identify the corresponding patients from the register that the specific surgeon had operated on, in addition to determining the surgical technique used for that specific patient. Therefore, the questionnaire was not answered anonymously by surgeons.

A total of 108 surgeons (61.7%) replied to the questionnaire [7]. From the results of the questionnaire, groups were created with specific combinations of surgical techniques of single-bundle ACL reconstruction based on eight relevant items selected from the questionnaire. Each group had a mandatory ‘Yes’ or ‘No’ answer requirement for certain items that subsequently identified that particular group (Table 2).

**Table 2:** Answer requirements characterizing defined groups

	Use of an Acc. medial portal	Visualization of the femoral ACL insertion site	Visualization of the tibial ACL insertion site	Lateral intercondylar ridge identified	Bifurcate ridge identified	Placing the femoral tunnel(s) in the femoral ACL insertion site	Placing the tibial tunnel(s) in the tibial ACL insertion site	Transportal drilling of the femoral ACL tunnel(s)
*Group*								
TP reference	Yes	Yes	Yes	Yes	Yes	Yes	Yes	Yes
TP anatomic						Yes	Yes	Yes
TT anatomic						Yes	Yes	No
TT partial-anatomic						No	Yes	No
TT non-anatomic						No	No	No
All landmarks		Yes	Yes	Yes	Yes			
No landmarks		No	No	No	No			
TP drilling								Yes
TT drilling								No

Empty spaces are not assigned a mandatory answer requirement. Surgeons can thus answer ‘Yes’ or ‘No’ to these items

*Acc* accessory, *TP* transportal, *TT* transtibial

### Outcome

Primary outcomes consisted of all sub-scales of the KOOS_4_. The KOOS is a knee-specific score, containing five sub-scales evaluating both the short- and long-term consequences of knee injuries, which includes post-traumatic osteoarthritis [31]. The KOOS_4_ is an average score of four KOOS sub-scales, in which function throughout daily living is excluded to avoid any ceiling effect due to the fact that relatively young and active patients rarely have difficulties with function in daily living [12].

### Statistical analysis

Tables were generated using Microsoft Word (Version 14.0.7, Microsoft Corp., Redmond, Washington, USA). A statistician assigned to the SNKLR performed all statistical analyses, which was undertaken using a standard statistical software package (SPSS Version 23.0, IBM Corp, Armonk, New York, USA). Data were characterized according to the level of measurement as nominal scale data, ordinal scale data, and ratio scale data. Means of normally distributed continuous data were compared with the independent-samples *t* test. Univariate ANOVA adjusted for age at index ACL reconstruction and gender was used to analyse the interaction of surgical technique and dimensions of the KOOS. Pairwise comparisons with *t* test were used to study differences between surgical techniques at pre-operative and 1- and 2-year follow-up (Supplementary file). All available patients with complete data at a single follow-up were included in the cross-sectional analyses. A general linear model was created by a mixed ANOVA with repeated measures to analysis the change in KOOS_4_ and the interaction with age at index ACL reconstruction, gender, and surgical techniques. Only patients with complete data from all three follow-ups were included in the repeated measures analysis. Mauchly’s test of sphericity was used to test the assumption of sphericity. If violated a Greenhouse–Geisser correction was used. Alpha was set to *p* < 0.05. An additional pairwise repeated measures analysis was conducted with a linear mixed model with fixed effects of age at index reconstruction, gender, and concomitant injuries, to account the occasional loss of KOOS_4_ data among patients at any point of follow-up.

## Results

**Fig. 1:**
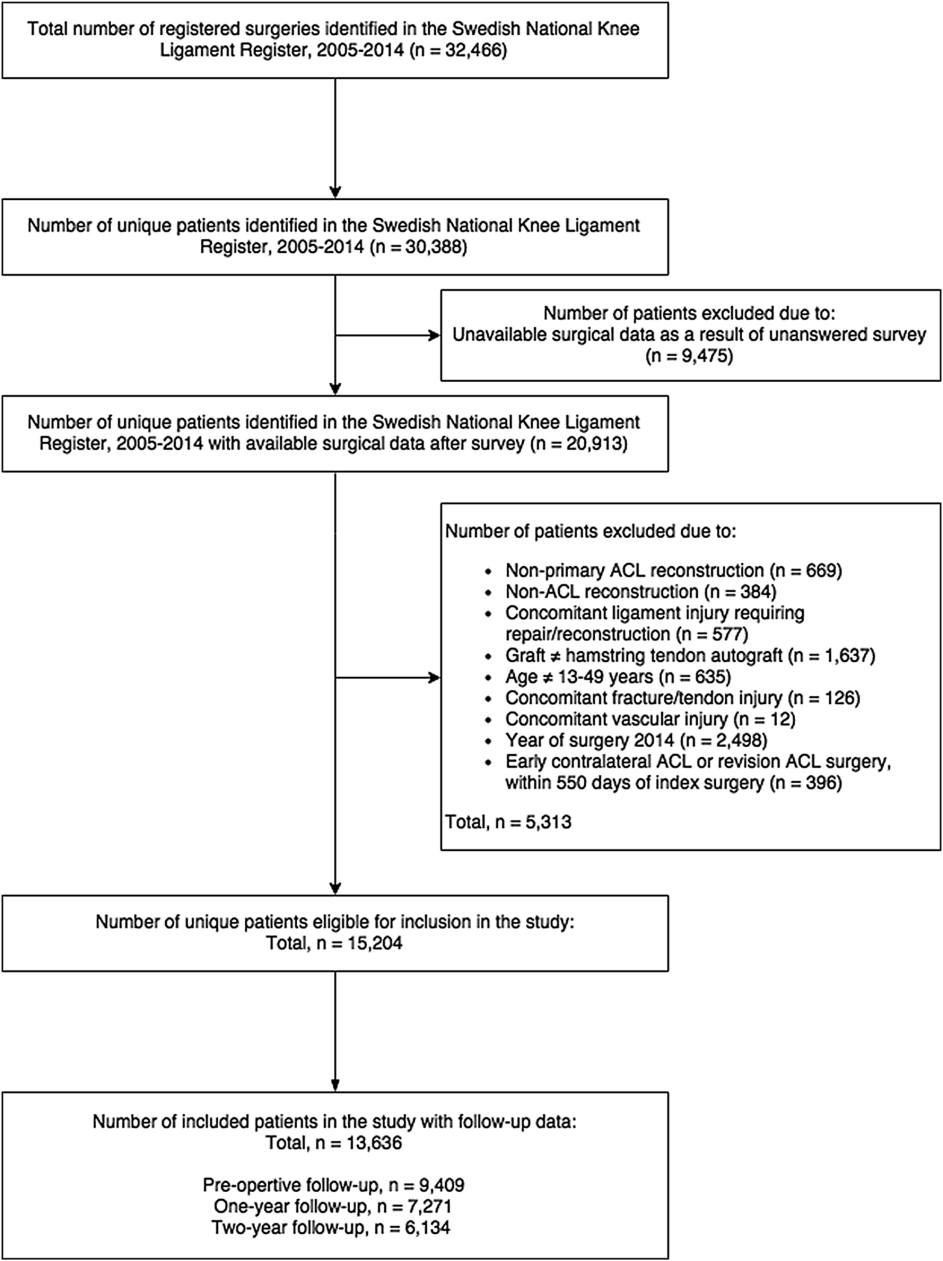
Flow chart demonstrating the selection of eligible patients from the Swedish National Knee Ligament Register

Data from 30,388 unique patients identified in the SNKLR between January 2005 and December 2014. Of these patients, a total of 20,913 were eligible for inclusion, and after applying all the exclusion criteria, data from 13,636 patients were included in the study (Fig. 1). Demographics of the included patients with complete data for the analyses are presented in Table 3.

**Table 3:** Patient characteristics at index anterior cruciate ligament reconstruction

Descriptive	Surgical technique *n* (%)					
	TP reference *n* = 5287 (34.8)	TT non-anatomic *n* = 1271 (8.4)	TT anatomic *n* = 1978 (13.0)	TT partial-anatomic *n* = 1492 (9.8)	TP anatomic *n* = 3608 (23.7)	Total cohort *n* = 13,636 (100)
*Gender*						
Female	2265 (42.8)	555 (43.7)	888 (44.9)	639 (42.8)	1579 (43.8)	5926 (43.5)
*Age at index ACL reconstruction*						
13–15	449 (8.5)	88 (6.9)	169 (8.5)	86 (5.8)	194 (5.4)	986 (7.2)
16–20	1480 (28.0)	401 (31.5)	586 (28.7)	392 (26.3)	1068 (29.6)	3909 (28.7)
21–25	1092 (20.7)	282 (22.2)	334 (16.9)	315 (21.1)	786 (21.8)	2809 (20.6)
26–30	799 (15.1)	162 (12.7)	260 (13.1)	232 (15.5)	515 (14.2)	1968 (14.4)
31–35	506 (9.6)	127 (10.0)	240 (12.1)	164 (11.0)	358 (9.9)	1395 (10.2)
36–40	462 (8.7)	107 (8.4)	198 (10.0)	169 (11.3)	303 (8.4)	1239 (9.2)
40–49	499 (9.4)	104 (8.2)	209 (10.6)	134 (9.0)	384 (10.6)	1330 (9.8)
Mean age (years)	25.3	26.3	26.5	26.3	26.0	
*Concomitant injury* (*yes*)						
Meniscus	2462 (46.6)	478 (37.6)	734 (37.1)	578 (38.7)	1615 (44.8)	5867 (43.0)
Cartilage	1454 (27.5)	411 (32.2)	491 (24.8)	346 (23.3)	823 (22.8)	3525 (25.9)

*ACL* anterior cruciate ligament

### Repeated measures analysis

**Fig. 2:**
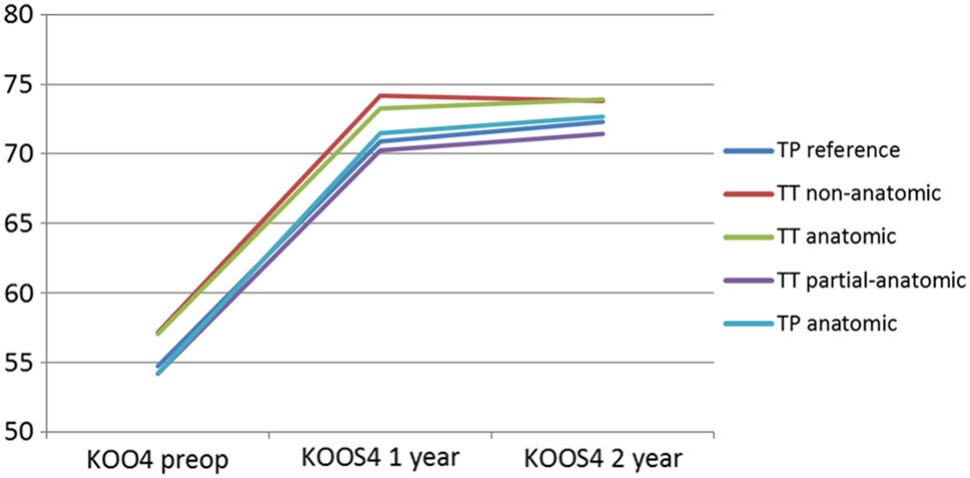
Average Knee injury and Osteoarthritis Outcome Score_4_ stratified by surgical technique pre-operatively and at 1- and 2-year follow-up after ACL reconstruction

A total of 2843 patients (1520 women and 1323 men) who had undergone single-bundle ACL reconstruction were included in the repeated measure analysis of KOOS_4_ from the pre-operative time point to follow-up at 2 years. Mauchly’s test of sphericity indicated that the assumption of sphericity had been violated, *χ* ^2^(2) = 338.678, (*p* < 0.001), and therefore, a Greenhouse–Geisser correction was used. A repeated measures ANOVA determined that no differences were found for the interaction between subjective knee function, KOOS_4_, and surgical techniques (Fig. 2). However, KOOS_4_ interaction with surgical technique significantly increased from pre-operatively to follow-up at 2 years (*p* = 0.006). Post hoc analysis of within group change revealed that KOOS_4_ significantly increased from pre-operatively to 1-year follow-up and pre-operative to 2-year follow-up for all surgical techniques of single-bundle ACL reconstruction (*p* < 0.001). However, no difference was found between 1- and 2-year follow-up (Table 4).

**Table 4:** Changes in Knee injury and Osteoarthritis Outcome Score_4_ from pre-operative to 2-year follow-up after ACL reconstruction

Surgical technique	KOOS_4_ pre-op	KOOS_4_ 1 year	KOOS_4_ 2 years	Mean improvement pre-op—1 year	*p* value	Mean improvement pre-op—2 years	*p* value	Mean improvement 1 year—2 years	*p* value
TP reference (*N* = 1097)	54.9	73.1	72.9	18.2	<0.001	17.9	<0.001	−0.2	n.s.
TP anatomic (*N* = 779)	54.4	72.6	73.0	18.2	<0.001	18.6	<0.001	0.4	n.s.
TT anatomic (*N* = 427)	56.2	74.3	73.6	18.1	<0.001	17.4	<0.001	−0.7	n.s
TT partial-anatomic (*N* = 322)	54.4	69.4	71.6	15.0	<0.001	17.2	<0.001	2.2	n.s.
TT non-anatomic (*N* = 298)	57.1	75.0	75.5	17.9	<0.001	18.4	<0.001	0.5	n.s.

*ACL* anterior cruciate ligament, *KOOS* Knee injury and Osteoarthritis Outcome Score, *TP* transportal, *TT* transtibial

### Linear mixed model analysis

The linear mixed model analysis accounted for the occasional loss of KOOS_4_ data among patients. In total, 12,133 unique patients were included in the analysis. No differences were found for the interaction of KOOS_4_ and surgical techniques of single-bundle ACL reconstruction with fixed adjustments for confounding factors, which included age at reconstruction, gender, and concomitant injuries (Table 5). Post hoc analysis of within group change revealed that KOOS_4_ significantly increased from pre-operatively to 1-year follow-up and pre-operative to 2-year follow-up for all surgical techniques of single-bundle ACL reconstruction (*p* < 0.001). No difference in KOOS_4_ was found between 1- and 2-year follow-up (Table 6).

**Table 5:** Linear mixed model on the interaction of KOOS_4_ and surgical technique of single-bundle ACL reconstruction adjusted for age at index reconstruction, gender, and concomitant injuries

Follow-up	Surgical technique^a^	Estimate	95% CI	*P* value
Pre-operative to one year	TP reference	−0.27	[−1.87; 1.33]	n.s.
	TP anatomic	0.94	[−0.74; 2.63]	n.s.
	TT anatomic	−0.004	[−1.87; 1.87]	n.s.
	TT partial-anatomic	−1.08	[−3.06; 0.89]	n.s.
One year to two years	TP reference	0.57	[−0.86; 2.01]	n.s.
	TP anatomic	0.76	[−0.74; 2.27]	n.s.
	TT anatomic	0.08	[−1.57; 1.74]	n.s.
	TT partial-anatomic	1.48	[−0.26; 3.22]	n.s.

*CI* confidence interval, *TP* transportal, *TT* transtibial

^a^TT non-anatomic set as reference

**Table 6:** Improvement in Knee injury and Osteoarthritis Outcome Score_4_ adjusted for age, sex, and concomitant injuries

Surgical technique	KOOS_4_ pre-op (*N* = 9409)	KOOS_4_ 1 year (*N* = 7271)	KOOS_4_ 2 years (*N* = 6134)	Mean improvement pre-op—1 year	*p* value	Mean improvement pre-op—2 years	*p* value	Mean improvement 1–2 years	*p* value
TP reference	53.6	70.1	70.8	16.5	<0.001	17.2	<0.001	0.7	n.s.
TP anatomic	52.9	70.6	71.5	17.7	<0.001	18.6	<0.001	0.9	n.s.
TT anatomic	55.7	72.5	72.6	16.8	<0.001	16.9	<0.001	0.1	n.s
TT partial-anatomic	53.1	68.8	70.4	15.7	<0.001	17.3	<0.001	1.6	n.s.
TT non-anatomic	55.9	72.7	72.8	16.8	<0.001	16.9	<0.001	0.1	n.s.

*ACL* anterior cruciate ligament, *KOOS* Knee injury and Osteoarthritis Outcome Score, *TP* transportal, *TT* transtibial

## Discussion

The main finding of this cohort study on patients after primary ACL reconstruction over a 10-year period was that surgical techniques of single-bundle reconstruction did not show differences in the change in KOOS_4_ during the first 2 years after surgery. Thus, the hypothesis was confirmed. However, subjective knee function as measured with KOOS_4_ improved for all surgical techniques from pre-operative to 2-year follow-up after ACL reconstruction.

Surgical techniques of single-bundle ACL reconstruction and optimal graft placement have been studied previously. As one example, grafts that are placed non-anatomically are exposed to lower forces [3, 18] and may explain reported differences in graft failure rates [7, 27]. Furthermore, the non-anatomic placement of a graft has the potential to result in residual rotational laxity of the knee, thus creating persisting instability [8] and potentially affecting subjective knee function. This cohort study intended to investigate how the technique of single-bundle ACL reconstruction affected subjective knee function one and 2 years after surgery compared to pre-operatively. No difference in improvement via the KOOS_4_ was found between the surgical techniques of single-bundle ACL reconstruction. Interestingly, patients who underwent ACL reconstruction with transtibial drilling of the femoral tunnel had a tendency towards superior results in the KOOS pre-operatively, as compared to transportal femoral tunnel drilling. Nevertheless, the repeated measures analyses showed that all surgical techniques of single-bundle ACL reconstruction had similar improvement in KOOS and equivalent results at 2 years after ACL reconstruction. Additionally, the post hoc pairwise comparison of all sub-scales of KOOS revealed that only one significant difference in the KOOS sub-scale of sport and recreation remained at 2-year follow-up; TT anatomic had a higher score than TT partial-anatomic (4.53, *p* = 0.006) (Supplementary file). This difference was, however, not clinically relevant [30].

The results of the study suggest that no clinically relevant differences are seen in subjective knee function with respect to surgical techniques of single-bundle reconstruction up to 2 years after surgery. Patient-reported knee function, such as the KOOS, has been suggested to provide an indirect evaluation of stability of the knee joint [33]. However, it cannot be ruled out that the KOOS is too non-specific to identify surgery-related differences in the knee joint and may not be an appropriate outcome for the evaluation of surgical techniques of ACL reconstruction. In comparison, an objective measure of knee stability, such as a quantifiable pivot shift test, can identify small differences in knee joint kinematics and may therefore be better suited as an outcome measurement in this scenario. However, no data on objective measures of knee stability are kept at follow-up in the SNKLR. Also of interest, the indication of the performed surgical technique and the potential learning curve after a particular technique is used for a period of time was not studied. Intuitively, it can be argued that if patients do not perceive a residual instability of the knee joint, this will limit the negative effect on subjective knee function [7, 8]. In addition, treatment failure in the form of early graft rupture was excluded in the study and may potentially have skewed the results of surgical techniques.

The general linear model created for the repeated measures analysis only included patients with complete KOOS_4_ data from all three time points of follow-up. The SNKLR has an insufficient response rate of 50–70% for the patient-reported outcome measures [10]. This led to a large number of patients being excluded from the initial analysis, potentially inflicting bias. A non-response analysis of the SNKLR has been conducted and reported that the SNKLR is valid despite the sub-optimal number of patients responding at follow-up [29]. Nevertheless, to increase the number of patients included in the data analyses, an additional repeated measures analysis was conducted based on a linear mixed model. The linear mixed model allows occasional loss of data unlike the general linear model, which means that the cohort is considerably larger and that estimates are more precise. Interestingly, no differences in the change in KOOS_4_ from baseline were found with any of repeated measure analyses.

In this study, a retrospective analysis was performed through a web-based questionnaire on surgical data, which, in turn, can entail an element of a recall bias. To minimize recall bias, responders were instructed to only answer the question if they were sure of the date (by specifying the year) that they adopted or abandoned the surgical technique in question. Moreover, all patients that were operated on during the time periods that the surgeon was ‘in-between’ surgical techniques were not included [7]. Further limitations in this study were that patient-specific data on activity level and rehabilitation were not reported in the registry. For example, objective measures of knee function, such as muscle strength, have been reported to explain a moderate proportion of the variation in patient-reported outcomes after ACL reconstruction [16]. Accordingly, an increased risk of graft failure has been reported when patients do not pass pre-defined goals in functional tests for return to activity and a decision to return to knee strenuous activity [15, 21]. On average, our results are in line with subjective knee function as measured with the KOOS and reported in the SNKLR [1, 13]. Nevertheless, the results are below the results that have been suggested as a functional recovery among patients after ACL reconstruction [5, 17]. Despite the exclusion of early graft failures, the proportion of patients not achieving an acceptable level of symptoms after treatment is not known in the cohort.

The strength of the present study is that it utilizes a large national registry, which is a unique source of information that consists of data from thousands of patients with a high follow-up rate from both patients and surgeons alike. In addition, to our knowledge, this is one of few studies that include detailed data of surgical technique to investigate patient-reported outcome after ACL reconstruction. Future research should aim to account for the potential confounding factors by including interdisciplinary data, e.g. from orthopaedic surgeons and physical therapists.

## Conclusion

In this study encompassing 13,636 patients from the SNKLR, the surgical technique of primary single-bundle ACL reconstruction did not demonstrate differences in improvement in baseline subjective knee function as measured with the KOOS_4_ at 2-year follow-up. However, subjective knee function improved from pre-operative baseline to 2-year follow-up independently of surgical technique, with the largest improvement seen in the first year after surgery.

## Electronic supplementary material

Below is the link to the electronic supplementary material.

Supplementary material 1 (DOCX 54 kb)
