# Supplementary material for: No differences in subjective knee function between surgical techniques of anterior cruciate ligament reconstruction at 2-year follow-up: a cohort study from the Swedish National Knee Ligament Register
Source: Knee Surg Sports Traumatol Arthrosc. 2017 Mar 17;25(12):3945–54. doi: 10.1007/s00167-017-4521-y (PMC5698359; doi:10.1007/s00167-017-4521-y)
Supplement: Supplementary file 1 — Supplementary material 1 (DOCX 54 kb) [file 167_2017_4521_MOESM1_ESM.docx]

## Cross Sectional Analysis

#### Pre-operative time point

| Table 4. Cross table of pairwise comparison of KOOS domains at preoperative follow up, stratified by Surgical Technique and adjusted for Age at index ACL reconstruction and Gender. | | | | | |
| --- | --- | --- | --- | --- | --- |
|  | **TP reference**  Mean difference  [95% CI]  P value | **TT non-anatomic**  Mean difference  [95% CI]  P value | **TT anatomic**  Mean difference  [95% CI]  P value | **TT partial-anatomic**  Mean difference  [95% CI]  P value | **TP anatomic**  Mean difference  [95% CI]  P value |
| **KOOS Pain** | | | | | |
| **TP reference**  n= 3,735  Mean 73.5 |  | -2.12  [-4.02; -0.22]  0.018 | -1.83  [-3.37; -0.30]  0.008 | -0.56  [-2.35; 1.22]  1.00 | -0.10  [-1.38; 1.18]  1.00 |
| **TT non-anatomic**  n= 787  Mean 76.2 | 2.12  [0.22; 4.02]  0.018 |  | 0.29  [-1.89; 2.46]  1.00 | 1.55  [-0.80; 3.91]  0.642 | 2.02  [0.02; 4.02]  0.046 |
| **TT anatomic**  n= 1,365  Mean 75.9 | 1.83  [0.30; 3.37]  0.008 | -0.29  [-2.46; 1.89]  1.00 |  | 1.27  [-0.81; 3.34]  0.86 | 1.73  [0.08; 3.38]  0.033 |
| **TT partial-anatomic**  n= 918  Mean 74.4 | 0.56  [-1.22; 2.35]  1.00 | -1.55  [-3.91; 0.80]  0.642 | -1.27  [-3.34; 0.81]  0.86 |  | 0.46  [-1.42; 2.35]  1.00 |
| **TP anatomic**  n= 2,357  Mean 74.2 | 0.10  [-1.18; 1.38]  1.00 | -2.02  [-4.02; -0.02]  0.046 | -1.73  [-3.38; -0.08]  0.033 | -0.46  [-2.35; 1.42]  1.00 |  |
| **KOOS Symptoms** | | | | | |
| **TP reference**  n= 3,734  Mean 69.5 |  | -1.34  [-3.36; 0.67]  0.611 | -1.95  [-3.58; -0.33]  0.008 | 0.64  [-1.25; 2.54]  1.00 | 0.18  [-1.18; 1.53]  1.00 |
| **TT non-anatomic**  n= 787  Mean 71.0 | 1.34  [-0.67; 3.36]  0.611 |  | -0.61  [-2.91; 1.69]  1.00 | 1.99  [-0.51; 4.48]  0.253 | 1.52  [-0.60; 3.63]  0.437 |
| **TT anatomic**  n= 1,365  Mean 71.6 | 1.95  [0.33; 3.58]  0.008 | 0.61  [-1.69; 2.91]  1.00 |  | 2.59  [0.40; 4.79]  0.009 | 2.13  [0.38; 3.88]  0.007 |
| **TT partial-anatomic**  n= 918  Mean 69.0 | -0.64  [-2.54; 1.25]  1.00 | -1.99  [-4.48; 0.51]  0.253 | -2.59  [-4.79; -0.40]  0.009 |  | -0.47  [-2.47; 1.53]  1.00 |
| **TP anatomic**  n= 2,357  Mean 69.4 | -0.18  [-1.53; 1.18]  1.00 | -1.52  [-3.63; 0.60]  0.437 | -2.13  [-3.88; -0.38]  0.007 | 0.47  [-1.53; 2.47]  1.00 |  |
| **KOOS ADL** | | | | | |
| **TP reference**  n= 3,735  Mean 82.7 |  | -2.87  [-4.67; -1.06]  < 0.001 | -2.66  [-4-12; -1.20]  < 0.001 | -1.47  [-3.17; 0.23]  0.148 | -0.58  [-1.79; 0.63]  1.00 |
| **TT non-anatomic**  n= 787  Mean 86.6 | 2.87  [1.06; 4.67]  < 0.001 |  | 0.21  [-1.86; 2.27]  1.00 | 1.39  [-0.84; 3.63]  0.80 | 2.29  [0.39; 4.18]  0.007 |
| **TT anatomic**  n= 1,365  Mean 85.4 | 2.66  [1.20; 4-12]  < 0.001 | -0.21  [-2.27; 1.86]  1.00 |  | 1.19  [-0.78; 3.15]  0.895 | 2.08  [0.51; 3.65]  0.002 |
| **TT partial-anatomic**  n= 918  Mean 84.2 | 1.47  [-0.23; 3.17]  0.148 | -1.39  [-3.63; 0.84]  0.80 | -1.19  [-3.15; 0.78]  0.895 |  | 0.89  [-0.90; 2.68]  1.00 |
| **TP anatomic**  n= 2,356  Mean 83.3 | 0.58  [-0.63; 1.79]  1.00 | -2.29  [-4.18; -0.39]  0.007 | -2.08  [-3.65; -0.51]  0.002 | -0.89  [-2.68; 0.90]  1.00 |  |
| **KOOS Sports** | | | | | |
| **TP reference**  n= 3,735  Mean 39.5 |  | -3.35  [-6.29; -0.41]  0.014 | -4.67  [-7.04; -2.30]  < 0.001 | 0.34  [-2.42; 3.10]  1.00 | 0.09  [-1.89; 2.07]  1.00 |
| **TT non-anatomic**  n= 787  Mean 42.0 | 3.35  [0.41; 6.29]  0.014 |  | -1.32  [-4.67; 2.04]  1.00 | 3.69  [0.05; 7.33]  0.044 | 3.45  [0.36; 6.53]  0.017 |
| **TT anatomic**  n= 1,365  Mean 44.3 | 4.67  [2.30; 7.04]  < 0.001 | 1.32  [-2.04; 4.67]  1.00 |  | 5.01  [1.81; 8.21]  < 0.001 | 4.76  [2.21; 7.32]  < 0.001 |
| **TT partial-anatomic**  n= 918  Mean 39.3 | -0.34  [-3.10; 2.42]  1.00 | -3.69  [-7.33; -0.05]  0.044 | -5.01  [-8.21; -1.81]  < 0.001 |  | -0.25  [-3.16; 2.67]  1.00 |
| **TP anatomic**  n= 2,356  Mean 39.5 | -0.09  [-2.07; 1.89]  1.00 | -3.45  [-6.53; -0.36]  0.017 | -4.76  [-7.32; -2.21]  < 0.001 | 0.25  [-2.67; 3.16]  1.00 |  |
| **KOOS QoL** | | | | | |
| **TP reference**  n= 3,735  Mean 32.9 |  | -1.74  [-3.77; 0.30]  0.165 | -0.78  [-2.42; 0.86]  1.00 | 0.92  [-0.99; 2.82]  1.00 | 1.13  [-0.24; 2.49]  0.206 |
| **TT non-anatomic**  n= 787  Mean 34.7 | 1.74  [-0.30; 3.77]  0.165 |  | 0.95  [-1.37; 3.28]  1.00 | 2.65  [0.14; 5.17]  0.031 | 2.86  [0.73; 5.00]  0.002 |
| **TT anatomic**  n= 1,365  Mean 33.7 | 0.78  [-0.86; 2.42]  1.00 | -0.95  [-3.28; 1.37]  1.00 |  | 1.70  [-0.51; 3.91]  0.31 | 1.91  [0.14; 3.67]  0.024 |
| **TT partial-anatomic**  n= 918  Mean 32.0 | -0.92  [-2.82; 0.99]  1.00 | -2.65  [-5.17; -0.14]  0.031 | -1.70  [-3.91; 0.51]  0.31 |  | 0.21  [-1.81; 2.22]  1.00 |
| **TP anatomic**  n= 2,356  Mean 31.8 | -1.13  [-2.49; 0.24]  0.206 | -2.86  [-5.00; -0.73]  0.002 | -1.91  [-3.67; -0.14]  0.024 | -0.21  [-2.22; 1.81]  1.00 |  |
| **KOOS_4_** | | | | | |
| **TP reference**  n= 3,734  Mean 54.1 |  | -2.15  [-4.02; -0.27]  0.013 | -2.30  [-3.81; -0.79]  < 0.001 | 0.34  [-1.42; 2.10]  1.00 | 0.33  [-0.93; 1.58]  1.00 |
| **TT non-anatomic**  n= 786  Mean 56.2 | 2.15  [0.27; 4.02]  0.013 |  | -0.16  [-2.30; 1.98]  1.00 | 2.48  [0.16; 4.81]  0.027 | 2.47  [0.50; 4.44]  0.004 |
| **TT anatomic**  n= 1,365  Mean 56.4 | 2.30  [0.79; 3.81]  < 0.001 | 0.16  [-1.98; 2.30]  1.00 |  | 2.64  [0.60; 4.68]  0.003 | 2.63  [1.00; 4.26]  < 0.001 |
| **TT partial-anatomic**  n= 918  Mean 53.7 | -0.34  [-2.10; 1.42]  1.00 | -2.48  [-4.81; -0.16]  0.027 | -2.64  [-4.68; -0.60]  0.003 |  | -0.01  [-1.87; 1.84]  1.00 |
| **TP anatomic**  n= 2,356  Mean 53.8 | -0.33  [-1.58; 0.93]  1.00 | -2.47  [-4.44; -0.50]  0.004 | -2.63  [-4.26; -1.00]  < 0.001 | 0.01  [-1.84; 1.87]  1.00 |  |

#### 1 year follow-up

| Table 5. Cross table of pairwise comparison of KOOS domains at 1 year follow up, stratified by Surgical Technique and adjusted for Age at index ACL reconstruction and Gender. | | | | | |
| --- | --- | --- | --- | --- | --- |
|  | **TP reference**  Mean difference  95% CI  P value | **TT non-anatomic**  Mean difference  95% CI  P value | **TT anatomic**  Mean difference  95% CI  P value | **TT partial-anatomic**  Mean difference  95% CI  P value | **TP anatomic**  Mean difference  95% CI  P value |
| **KOOS Pain** | | | | | |
| **TP reference**  n= 2,877  Mean 84.0 |  | -2.04  [-3.79; -0.28]  0.011 | -1.37  [-3.00; 0.26]  0.185 | 0.56  [-1.15; 2.27]  1.00 | -0.57  [-1.94; 0.80]  1.00 |
| **TT non-anatomic**  n= 805  Mean 86.0 | 2.04  [0.28; 3.79]  0.011 |  | 0.67  [-1.43; 2.77]  1.00 | 2.60  [0.44; 4.76]  0.007 | 1.47  [-0.43; 3.37]  0.297 |
| **TT anatomic**  n= 972  Mean 85.4 | 1.37  [-0.26; 3.00]  0.185 | -0.67  [-2.77; 1.43]  1.00 |  | 1.93  [-0.13; 3.99]  0.084 | 0.80  [-0.98; 2.59]  1.00 |
| **TT partial-anatomic**  n= 863  Mean 83.4 | -0.56  [-2.27; 1.15]  1.00 | -2.60  [-4.76; -0.44]  0.007 | -1.93  [-3.99; 0.13]  0.084 |  | -1.13  [-2.99; 0.73]  0.872 |
| **TP anatomic**  n= 1,620  Mean 84.6 | 0.57  [-0.80; 1.95]  1.00 | -1.47  [-3.37; 0.43]  0.297 | -0.80  [-2.58; 0.98]  1.00 | 1.13  [-0.73; 2.99]  0.872 |  |
| **KOOS Symptoms** | | | | | |
| **TP reference**  n= 2,877  Mean 76.7 |  | -3.56  [-5.54; -1.55]  < 0.001 | -2.18  [-4.04; -0.33]  0.01 | 0.01  [-1.93; 1.96]  1.00 | -0.50  [-2.06; 1.06]  1.00 |
| **TT non-anatomic**  n= 805  Mean 80.2 | 3.56  [1.55; 5.54]  < 0.001 |  | 1.36  [-1.03; 3.75]  1.00 | 3.56  [1.11; 6.01]  < 0.001 | 3.05  [0.89; 5.21]  0.001 |
| **TT anatomic**  n= 972  Mean 78.9 | 2.18  [0.33; 4.04]  0.01 | -1.36  [-3.75; 1.03]  1.00 |  | 2.19  [-0.15; 4.54]  0.086 | 1.68  [-0.35; 3.72]  0.202 |
| **TT partial-anatomic**  n= 862  Mean 76.7 | -0.01  [-1.96; 1.93]  1.00 | -3.56  [-6.01; -1.11]  < 0.001 | -2.19  [-4.54; 0.15]  0.086 |  | -0.51  [-2.62; 1.60]  1.00 |
| **TP anatomic**  n= 1,619  Mean 77.2 | 0.50  [-1.06; 2.06]  1.00 | -3.05  [-5.21; -0.89]  0.001 | -1.68  [-3.72; 0.35]  0.202 | 0.51  [-1.60; 2.62]  1.00 |  |
| **KOOS ADL** | | | | | |
| **TP reference**  n= 2,875  Mean 91.0 |  | -1.83  [-3.27; -0.39]  0.003 | -1.46  [-2.80; -0.12]  0.022 | 0.21  [-1.19; 1.60]  1.00 | -0.14  [-1.26; 0.98]  1.00 |
| **TT non-anatomic**  n= 805  Mean 92.8 | 1.83  [0.39; 3.27]  0.003 |  | 0.37  [-1.35; 2.09]  1.00 | 2.04  [0.27; 3.81]  0.012 | 1.68  [0.14; 3.50]  0.022 |
| **TT anatomic**  n= 972  Mean 92.5 | 1.46  [0.12; 2.80]  0.022 | -0.37  [-2.09; 1.35]  1.00 |  | 1.67  [-0.02; 3.36]  0.055 | 1.32  [-0.14; 2.79]  0.112 |
| **TT partial-anatomic**  n= 864  Mean 90.8 | -0.21  [-1.60; 1.19]  1.00 | -2.04  [-3.81; -0.27]  0.012 | -1.67  [-3.36; 0.02]  0.055 |  | -0.35  [-1.86; 1.17]  1.00 |
| **TP anatomic**  n= 1,620  Mean 91.1 | 0.14  [-0.98; 1.26]  1.00 | -1.68  [-3.50; -0.14]  0.022 | -1.32  [-2.79; 0.14]  0.112 | 0.35  [-1.17; 1.86]  1.00 |  |
| **KOOS Sports** | | | | | |
| **TP reference**  n= 3,735  Mean 73.5 |  | -3.59  [-6.58; -0.59]  0.008 | -3.65  [-6.43; -0.86]  0.002 | 1.68  [-1.23; 4.59]  1.00 | -1.12  [-3.45; 1.42]  1.00 |
| **TT non-anatomic**  n= 787  Mean 76.2 | 3.59  [0.59; 6.58]  0.008 |  | -0.06  [-3.64; 3.52]  1.00 | 5.27  [1.59; 8.94]  < 0.001 | 2.46  [-0.77; 5.70]  0.325 |
| **TT anatomic**  n= 1,365  Mean 75.9 | 3.65  [0.86; 6.43]  0.002 | 0.06  [-3.52; 3.64]  1.00 |  | 5.33  [1.82; 8.84]  0.001 | 2.53  [-0.52; 5.57]  0.200 |
| **TT partial-anatomic**  n= 918  Mean 74.4 | -1.68  [-4.59; 1.23]  1.00 | -5.27  [-8.94; -1.59]  < 0.001 | -5.33  [-8.84; 1.82]  0.001 |  | -2.80  [-5.96; 0.36]  0.128 |
| **TP anatomic**  n= 2,357  Mean 74.2 | 1.12  [-1.42; 3.45]  1.00 | -2.46  [-5.70 ;0.77]  0.325 | -2.53  [-5.57; 0.52]  0.200 | 2.80  [-0.36; 5.96]  0.128 |  |
| **KOOS QoL** | | | | | |
| **TP reference**  n= 2,877  Mean 59.0 |  | -3.95  [-6.60; -1.30]  < 0.001 | -2.91  [-5.38; -0.44]  0.009 | 0.27  [-2.31; 2.85]  1.00 | -1.07  [-3.14; 0.99]  1.00 |
| **TT non-anatomic**  n= 805  Mean 62.9 | 3.95  [1.30; 6.60]  < 0.001 |  | 1.04  [-2.12; 4.21]  1.00 | 4.22  [0.97; 7.47]  0.003 | 2.87  [0.01; 5.74]  0.049 |
| **TT anatomic**  n= 972  Mean 61.9 | 2.91  [0.44; 5.38]  0.009 | -1.04  [-4.21; 2.12]  1.00 |  | 3.18  [0.08; 6.29]  0.040 | 1.84  [-0.86; 4.53]  0.56 |
| **TT partial-anatomic**  n= 865  Mean 58.7 | -0.27  [-2.85; 2.31]  1.00 | -4.22  [-7.47; -0.97]  0.003 | -3.18  [-6.29; -0.08]  0.040 |  | -1.35  [-4.14; 1.45]  1.00 |
| **TP anatomic**  n= 1,620  Mean 60.1 | 1.07  [-0.99; 3.14]  1.00 | -2.87  [-5.74; -0.01]  0.049 | -1.84  [-4.53; 0.86]  0.56 | 1.35  [-1.45; 4.14]  1.00 |  |
| **KOOS_4_** | | | | | |
| **TP reference**  n= 2,876  Mean 71.0 |  | -3.28  [-5.40; -1.17]  < 0.001 | -2.52  [-4.49; -0.55]  0.003 | 0.58  [-1.49; 2.63]  1.00 | -0.60  [-2.45; 0.84]  1.00 |
| **TT non-anatomic**  n= 805  Mean 72.3 | 3.28  [1.17; 5.40]  < 0.001 |  | 0.76  [-1.77; 3.29]  1.00 | 3.86  [1.26; 6.45]  < 0.001 | 2.48  [0.19; 4.76]  0.023 |
| **TT anatomic**  n= 971  Mean 73.5 | 2.52  [0.55; 4.49]  0.003 | -0.76  [-3.29; 1.77]  1.00 |  | 3.09  [0.61; 5.58]  0.003 | 1.72  [-0.44; 3.87]  0.253 |
| **TT partial-anatomic**  n= 861  Mean 70.4 | -0.58  [-2.63; 1.49]  1.00 | -3.86  [-6.45; -1.26]  < 0.001 | -3.09  [-5.58; -0.61]  0.003 |  | -1.38  [-3.61; 0.86]  0.834 |
| **TP anatomic**  n= 1,619  Mean 71.8 | 0.60  [-0.84; 2.45]  1.00 | -2.48  [-4.76; -0.19]  0.023 | -1.72  [-3.87; 0.44]  0.253 | 1.38  [-0.86; 3.61]  0.834 |  |

#### 2 Year Follow up

| Table 6. Cross table of pairwise comparison of KOOS sub-scales at 2 year follow-up, stratified by Surgical Technique and adjusted for Age at index ACL reconstruction and Gender. | | | | | |
| --- | --- | --- | --- | --- | --- |
|  | **TP reference**  Mean difference  95% CI  P value | **TT non-anatomic**  Mean difference  95% CI  P value | **TT anatomic**  Mean difference  95% CI  P value | **TT partial-anatomic**  Mean difference  95% CI  P value | **TP anatomic**  Mean difference  95% CI  P value |
| **KOOS Pain** | | | | | |
| **TP reference**  n= 2,258  Mean 84.8 |  | -1.10  [-3.13; 0.93]  1.00 | -1.24  [-2.93; 0.45]  0.388 | 0.10  [-1.78; 1.99]  1.00 | -0.86  [-2.31; 0.59]  0.97 |
| **TT non-anatomic**  n= 581  Mean 85.9 | 1.10  [-0.93; 3.13]  1.00 |  | -0.14  [-2.44; 2.13]  1.00 | 1.20  [-1.24; 3.65]  1.00 | 0.24  [-1.88; 2.37]  1.00 |
| **TT anatomic**  n= 944  Mean 86.1 | 1.24  [-0.45; 2.93]  0.388 | 0.14  [-2.13; 2.44]  1.00 |  | 1.35  [-0.83; 3.52]  0.818 | 0.39  [-1.42; 2.20]  1.00 |
| **TT partial-anatomic**  n= 702  Mean 84.7 | -0.10  [-1.99; 1.78]  1.00 | -1.20  [-3.65; 1.24]  1.00 | -1.35  [-3.52; 0.83]  0.818 |  | -0.96  [-2.95; 1.03]  1.00 |
| **TP anatomic**  n= 1,503  Mean 85.7 | 0.86  [-0.59; 2.31]  0.97 | -0.24  [-2.37; 1.88]  1.00 | -0.39  [-2.20; 1.42]  1.00 | 0.96  [-1.03; 2.95]  1.00 |  |
| **KOOS Symptoms** | | | | | |
| **TP reference**  n= 2,257  Mean 78.6 |  | -1.43  [-3.74; 0.89]  0.84 | -0.93  [-2.86; 1.00]  1.00 | 0.10  [-2.05; 2.25]  1.00 | -0.68  [-2.34; 0.98]  1.00 |
| **TT non-anatomic**  n= 581  Mean 80.0 | 1.43  [-0.89; 3.74]  0.84 |  | 0.50  [-2.13; 3.13]  1.00 | 1.53  [-1.26; 4.32]  1.00 | 0.75  [-1.68; 3.18]  1.00 |
| **TT anatomic**  n= 944  Mean 79.5 | 0.93  [-1.00; 2.86]  1.00 | -0.50  [-3.13; 2.13]  1.00 |  | 1.03  [-1.45; 3.51]  1.00 | 0.25  [-1.82; 2.32]  1.00 |
| **TT partial-anatomic**  n= 702  Mean 78.5 | -0.10  [-2.25; 2.05]  1.00 | -1.53  [-4.32; 1.26]  1.00 | -1.03  [-3.51; 1.45]  1.00 |  | -0.78  [-3.05; 1.50]  1.00 |
| **TP anatomic**  n= 1,503  Mean 79.3 | 0.68  [-0.98; 2.34]  1.00 | -0.75  [-3.18; 1.68]  1.00 | -0.25  [-2.32; 1.82  1.00 | 0.78  [-1.50; 3.05]  1.00 |  |
| **KOOS ADL** | | | | | |
| **TP reference**  n= 2,257  Mean 91.3 |  | -1.51  [-3.21; 0.19]  0.125 | -1.13  [-2.54; 0.29]  0.254 | 0.72  [-0.86; 2.30]  1.00 | -0.50  [-0.19; 3.21]  1.00 |
| **TT non-anatomic**  n= 581  Mean 92.8 | 1.51  [-0.19; 3.21]  0.125 |  | 0.39  [-1.54; 2.31]  1.00 | 2.23  [0.19; 4.28]  0.022 | 1.01  [-0.78; 2.80]  1.00 |
| **TT anatomic**  n= 944  Mean 92.4 | 1.13  [-0.29; 2.54]  0.254 | -0.39  [-2.31; 1.54]  1.00 |  | 1.85  [0.03; 3.67]  0.044 | 0.62  [-0.89; 2.14]  1.00 |
| **TT partial-anatomic**  n= 702  Mean 90.6 | -0.72  [-2.30; 0.86]  1.00 | -2.23  [4.28; 0.19;]  0.022 | -1.85  [3.67; 0.03]  0.044 |  | -1.22  [-2.89; 0.45]  0.395 |
| **TP anatomic**  n= 1,502  Mean 91.8 | 0.50  [-3.21; 0.19]  1.00 | -1.01  [-2.80; 0.78;]  1.00 | -0.62  [-2.14; 0.89]  1.00 | 1.22  [-0.45; 2.89]  0.395 |  |
| **KOOS Sports** | | | | | |
| **TP reference**  n= 2,258  Mean 66.4 |  | -2.10  [-5.57; 1.38]  0.901 | -2.82  [-5.71; 0.08]  0.062 | 1.71  [-1.51; 4.94]  1.00 | -0.63  [-3.12; 1.86]  1.00 |
| **TT non-anatomic**  n= 581  Mean 68.5 | 2.10  [-1.38; 5.57]  0.901 |  | -0.72  [-4.66; 3.22]  1.00 | 3.81  [-0.38; 8.00]  0.106 | 1.47  [-2.18; 5.12]  1.00 |
| **TT anatomic**  n= 944  Mean 69.3 | 2.82  [-0.08; 5.71]  0.062 | 0.72  [-3.22; 4.66]  1.00 |  | 4.53  [0.81; 8.26]  0.006 | 2.19  [-0.91; 5.29]  0.477 |
| **TT partial-anatomic**  n= 702  Mean 64.7 | -1.71  [-4.94; 1.51]  1.00 | -3.81  [-8.00; 0.38]  0.106 | -4.53  [-8.26; -0.81]  0.006 |  | -2.34  [-5.76; 2.18]  0.538 |
| **TP anatomic**  n= 1,503  Mean 67.1 | 0.63  [-1.86; 3.12]  1.00 | -1.47  [-5.12; 2.18]  1.00 | -2.19  [-5.29; 0.91]  0.477 | 2.34  [-2.18; 5.76]  0.538 |  |
| **KOOS QoL** | | | | | |
| **TP reference**  n= 2,257  Mean 61.9 |  | -2.29  [-5.38; 0.80]  0.378 | -1.34  [-3.92; 1.23]  1.00 | 1.37  [-1.50; 4.24]  1.00 | -0.30  [-2.51; 1.91]  1.00 |
| **TT non-anatomic**  n= 581  Mean 64.1 | 2.29  [-0.80; 5.38]  0.378 |  | 0.95  [-2.56; 4.45]  1.00 | 3.66  [-0.07; 7.38]  0.058 | 1.99  [-1.26; 5.23]  0.857 |
| **TT anatomic**  n= 944  Mean 63.2 | 1.34  [-1.23; 3.92]  1.00 | -0.95  [-4.45; 2.56]  1.00 |  | 2.71  [-0.60; 6.02]  0.215 | 1.04  [-1.72; 3.80]  1.00 |
| **TT partial-anatomic**  n= 702  Mean 60.5 | -1.37  [-4.24; 1.50]  1.00 | -3.66  [-7.38; 0.07]  0.058 | -2.71  [-6.02; 0.60]  0.215 |  | -1.67  [-4.71; 1.36]  1.00 |
| **TP anatomic**  n= 1,503  Mean 62.2 | 0.30  [-1.91; 2.51]  1.00 | -1.99  [-5.23; 1.26]  0.857 | -1.04  [-3.80; 1.72]  1.00 | 1.67  [-1.36; 4.71]  1.00 |  |
| **KOOS_4_** | | | | | |
| **TP reference**  n= 2,256  Mean 72.9 |  | -1.74  [-4.21; 0.74]  0.485 | -1.59  [-3.65; 0.47]  0.300 | 0.81  [-1.48; 3.11]  1.00 | -0.62  [-2.39; 1.15]  1.00 |
| **TT non-anatomic**  n= 581  Mean 74.7 | 1.74  [-0.74; 4.21]  0.485 |  | 0.15  [-2.66; 2.95]  1.00 | 2.55  [-0.42; 5.53]  0.162 | 1.11  [-1.48; 3.71]  1.00 |
| **TT anatomic**  n= 944  Mean 74.5 | -1.59  [-0.47; 3.65]  0.300 | -0.15  [-2.95; 2.66]  1.00 |  | 2.40  [-0.24; 5.05]  0.108 | 0.97  [-1.24; 3.17]  1.00 |
| **TT partial-anatomic**  n= 702  Mean 72.1 | -0.81  [-3.11; 1.48]  1.00 | -2.55  [-5.53; 0.42]  0.162 | -2.40  [-5.05; 0.24]  0.108 |  | -1.44  [-3.86; 0.99]  0.961 |
| **TP anatomic**  n= 1,503  Mean 73.6 | 0.62  [-1.15; 2.39]  1.00 | -1.11  [-3.71; 1.48]  1.00 | -0.97  [-3.17; 1.24]  1.00 | 1.44  [-0.99; 3.86]  0.961 |  |
